# Abdominal Etching: A 10 Year Retrospective Study

**DOI:** 10.1093/asjof/ojaf018.004

**Published:** 2025-05-13

**Authors:** Dieter Brummund, Tarik Husain

**Affiliations:** Larkin Community Hospital, Miami, FL; Private Practice, Miami Beach, FL

## Abstract

**Goals/Purpose:**

Differential liposuction with abdominal etching is a powerful liposculpture technique to achieve a defined athletic aesthetic. Etching consists of selective superficial lipoaspiration to create adhesions mimicking dermal-fascial inscriptions. It can be performed alone, or in combination with energy or excisional contouring. This case series describes the evolution of the abdominal etching technique in the senior author's practice and evaluates outcomes.

**Methods/Technique:**

A retrospective chart review of 345 patients undergoing differential liposuction with abdominal etching between 2014 to 2024 by the senior author. The preoperative evaluation, markings, surgical technique and postoperative care are discussed. Clinical parameters assessed include age, gender, body mass index, combination of procedures including fat grafting, energy or excisional procedures, and volume of lipoaspirate. Outcomes evaluated include patient satisfaction and revision rate, minor complications including contour irregularity, hyperpigmentation, paresthesias, seroma, hematoma, cellulitis and burns, and major complications including deep venous thrombosis, pulmonary embolism, fat embolism, and unplanned hospitalization.

**Results/Complications:**

A total of 345 patients were identified with 124 males and 221 females. The mean age was 38.7 (range, 18 to 72 years), with a non obese, average body mass index of 27.67 kg/m2 (range, 17.85 to 41.6 kg/m2). 281 patients (81.44%) were primary lipocontouring and and 64 patients (18.55%) were secondary liposuction. 211 patients (61.16%) were treated with liposuction alone, 134 patients (38.84%) were combined with helium plasma radiofrequency, 115 patients with fat grafting and 51 patients (14.78%) with abdominoplasty. Average lipoaspirate was 3485 ml (range 200 - 9000 ml). Average energy delivered was 11.51 kJ (range 2 - 26 kJ), with a power setting of 80% (range 80 - 85%) and a flow rate of 2.71 L/min (range 2 - 3.5 L/min). Fat grafted regions included buttocks in 94 patients (81.73%), deltoid 7 (6.08%), chest 5 (4.34%), bicep 5 (4.34%), breast 4 (3.48%), calves 1 (0.87%).

328 patients (95.07%) were satisfied with their result. Of the 19 dissatisfied patients, 1 (5.26%) opted to not undergo a revision, 15 (78.84%) were satisfied following one revisionary procedure, 2 (10.52%) were satisfied following two revisionary procedures, and 1 (5.26%) was satisfied following three revisionary procedures. Minor complications included 34 patients (9.85%) with contour irregularity, 28 patients with seroma (8.11%), 19 patients with hyperpigmentation (5.50%), 12 patients (2.89%) with a surgical site infection managed with oral antibiotics, 3 patients (0.87%) with hematoma, 3 patients (0.87%) with paresthesias, and 2 patients (0.5%) with subdermal burns. Major complications included 1 patient with deep venous thrombosis and pulmonary embolism and 1 patient with negative pressure pulmonary edema following laryngospasm. There were no cases of fat embolism. Subgroup analysis revealed a trend toward an increased seroma rate with the combination of abdominal etching with abdominoplasty (n=7, 13.75%) and abdominal etching with helium radiofrequency (n=14, 10.37%) when compared with abdominal etching alone (n=14, 4.28%), however these were not statistically significant (p=0.07, p=0.06).

**Conclusion:**

Differential liposuction with abdominal etching is a safe and effective liposculpture technique to achieve an athletic physique and enhance abdominal aesthetics. It can be performed alone, or in combination with helium plasma radiofrequency or abdominoplasty. Complication rates are low and manageable with appropriate surgical technique. Almost all patients reviewed were satisfied with the procedure and maintained long-term results.

